# Application of the automated haematology analyzer XN-30 in an experimental rodent model of malaria

**DOI:** 10.1186/s12936-018-2313-6

**Published:** 2018-04-16

**Authors:** Takahiro Tougan, Yuhgi Suzuki, Munehisa Izuka, Kei Aono, Tomonori Okazaki, Yuji Toya, Kinya Uchihashi, Toshihiro Horii

**Affiliations:** 1 Department of Molecular Protozoology, Research Institute for Microbial Diseases, Osaka University, 3-1 Yamadaoka, Suita, Osaka 565-0871 Japan; 2 Sysmex Corporation, 4-4-4 Takatsukadai Nishiku, Kobe, Hyogo 651-2271 Japan

**Keywords:** Rodent malaria parasite, Mouse, Blood stage, Parasitaemia, Haematological state, Howell–Jolly body, Anti-malarial drug, Flow cytometry, XN-30 analyzer

## Abstract

**Background:**

The erythrocytic stage, where malaria parasites proliferate in human blood, is clinically significant as this causes the symptoms and illness of malaria. Experimental rodent models of malaria at the erythrocytic stage are used for the development of anti-malarial drugs and for biological analysis. An automated haematology analyzer XN-30 was developed for detection of infected red blood cells (iRBCs) in human blood samples and measurement of their parasitaemia in approximately 1 min through flow cytometry analysis. Additionally, the analyzer simultaneously measured other haematological parameters in these samples. It is inferred that the analyzer would also allow easy and rapid measurement of parasitaemia in mice and provide important clues on the mouse haematological state during infection and treatment.

**Results:**

The XN-30 analyzer is a simple and rapid tool to detect iRBCs in mouse blood samples infected with rodent malarial parasites, with three-dimensional analysis permitting the precise measurement of parasitaemia (referred herein as the ‘XN-30 system’). The XN-30 analyzer allowed not only the detection of iRBCs but also the monitoring of RBC, white blood cell, and platelet counts, as well as haematocrit, mean corpuscular volume and mean platelet volume values in the mouse blood sample. For anti-malarial drug development, aside from demonstrating possible efficacy in mouse models, XN-30 analyzer could provide a first glimpse of the safety profile of the drug.

**Conclusions:**

The XN-30 system is a powerful tool that can be utilized for the in vivo screening, development, and evaluation of anti-malarial drugs as well as for pre-clinical pharmacology and/or toxicity tests in rodent models.

**Electronic supplementary material:**

The online version of this article (10.1186/s12936-018-2313-6) contains supplementary material, which is available to authorized users.

## Background

The malarial parasite is a unicellular eukaryote that infects humans via mosquitoes. The erythrocytic stage of the parasite in humans is clinically significant as this stage of the *Plasmodium* parasite causes the symptoms of malaria. The disease symptoms can be very mild and uncomplicated to include fever or become severe progressing to anaemia, splenomegaly, and sometimes death. Various anti-malarial drugs, such as chloroquine and artemisinin, have targeted this stage of the parasite life cycle. Since *Plasmodium* species that infect humans are essentially unable to infect non-primate animals, rodent malarial parasites are typically utilized to evaluate the efficacy of anti-malarial compounds in vivo. Of these parasitic species, *Plasmodium berghei*, *Plasmodium yoelii*, *Plasmodium chabaudi*, and *Plasmodium vinckei* have been extensively used in drug discovery and early development [1]. In these drug discovery and development processes, microscopic analysis of Giemsa-stained blood smears is currently the standard method for measuring parasitaemia in rodent models of malaria. However, since this method is labour-intensive, time-consuming, and low-throughput [2], alternative methods, including flow cytometry, have been developed [3–6].

From the end user side, without requiring technical experience or expertise, an automated haematology analyzer XN-30 (Sysmex, Kobe, Japan) was developed to detect the malaria parasite and to calculate its parasitaemia in human blood samples in approximately 1 min through flow cytometry analysis [7]. In brief, the XN-30 analyzer aspirates and dilutes blood samples in a diluent solution (CELLPACK DCL) at a certain dilution ratio. Subsequently, the nucleic acids are stained with a staining solution (Fluorocell M) along with a lysis solution (Lysercell M) and infected red blood cells (iRBCs) and white blood cells (WBCs) are detected by a blue semiconductor 405 nm laser beam. At the same time, a sheath flow direct current is adopted for measuring 10 haematological parameters including RBC and platelet (PLT) counts, and haematocrit (HCT), mean corpuscular volume (MCV), and mean platelet volume (MPV) values [7]. The minimum detection sensitivity of the XN-30 analyzer in the whole blood mode, which allows quantitative counting of infected RBCs is 18 iRBCs/μL [7]. In addition, a new algorithm for in vitro cultured parasites was developed, which distinguished the different developmental stages of in vitro cultured *Plasmodium falciparum* parasite [8]. Thus, it was inferred that the XN-30 analyzer may be suitable not only for easy and rapid parasitaemia measurements in human blood or in vitro parasite culture but could also be an important experimental tool for in vivo drug screening and development in rodent malarial models.

The present study demonstrates that the XN-30 analyzer distinguishes iRBCs from WBCs, polychromatic RBCs, Howell-Jolly body-containing RBCs (HJB-RBCs), and merozoites through three-dimensional analysis using parameters of side scattered light (SSC, representing the internal cell structure and its contents), forward scattered light (FSC, indicating iRBC size), and side fluorescent light (SFL, corresponding to DNA content). In addition, the XN-30 analyzer can simultaneously monitor the following haematological parameters: RBC, WBC, and PLT counts, and HCT, MCV, and MPV values. Finally, the XN-30 system allowed the measurement of parasitaemia after treatment with the anti-malarial drug, artemisinin. XN-30 analyses after drug treatment could help not only to elucidate the pharmacokinetics of the drug but also monitor safety of the treatment and development of drug resistance.

## Methods

### Mice, parasites, and infection

C57BL/6 and ICR mice (6 weeks old) were purchased from Japan SLC, Inc. (Shizuoka, Japan). For parasite infection, the mice were injected intraperitoneally with 3 × 10^5^ iRBCs with the non-lethal strain, *P. yoelii* 17XNL, suspended in phosphate-buffered saline (PBS).

### Measurement of parasitaemia

Peripheral blood was collected from the tail vein in Na-EDTA-containing vials. The blood samples were analysed simultaneously using the XN-30 analyzer with the algorithm for cultured *P. falciparum* parasites [prototype; software version 01-03 (build 16)] (Sysmex) and by light microscopy using thin Giemsa-stained blood smears. Peripheral blood samples were diluted with PBS and applied to a Capiject Capillary Blood Collection Tube (Terumo, Tokyo, Japan). The samples were loaded onto the XN-30 analyzer as per the manufacturer’s instructions. The dedicated reagents, CELLPACK DCL, SULFOLYSER, Lysercell M, and Fluorocell M, were used for this measurement (Sysmex).

Standard thin blood smears were fixed in 100% methanol (Nacalai Tesque, Kyoto, Japan) for 10 min and then stained in 10% Giemsa stain working solution, pH 7.2 (Merck KGaA, Darmstadt, Germany) for 13 min. The slides were observed at 1000× magnification using a BX50 light microscope (Olympus, Tokyo, Japan). Parasitaemia was determined by calculating the ratio of iRBCs in at least 3000 RBCs.

### Data analysis by Flowing software

FCS files exported from the XN-30 analyzer were analysed by Flowing software 2.5.1 (Turku Centre for Biotechnology, University of Turku, Turku, Finland). Areas of iRBCs, polychromatic RBCs, HJB-RBCs, and WBCs were gated on the M scattergrams and areas of iRBCs, HJB-RBCs, and merozoites were gated on the M(SSC-FSC) scattergrams. Common dots in iRBC areas on both the M and M(SSC-FSC) scattergrams were assigned as iRBCs and counted (see Fig. 2b; red dots). This method using SFL and FSC parameters for M scattergrams, and SSC and FSC parameters for M(SSC-FSC) scattergrams was designated as ‘three-dimensional analysis’. Each scattergram represented dots per 0.953 μL and the analyzer reported the number of total RBCs per μL. This difference was compensated in the calculation of parasitaemia, as shown in following equation:

$$ {\text{Parasitaemia }}\left( \% \right)\, = \,{\text{iRBC count }}({\text{counts}}/0. 9 5 3 \,{\upmu\text{L}})/0. 9 5 3/{\text{total RBC count }}({\text{counts}}/{\upmu\text{L}})\, \times \, 100. $$

RBC, WBC, and PLT counts, as well as HCT value were calculated according to the indicated dilution ratio; MCV and MPV values were directly adopted.

The re-analysis of the scattergrams from the XN-30 analyzer by using Flowing software is referred to as the ‘XN-30 system’ in this study.

### Drug treatment analysis

As stock solution, artemisinin (TCI, Tokyo, Japan) was dissolved at 50 mg/mL in 65% dimethyl sulphoxide and 35% Tween-80. The stock solution was further diluted to 5 mg/mL (1:10 dilution) with saline (Otsuka Normal Saline; Otsuka Pharmaceutical Co Ltd, Tokyo, Japan). Mice were subcutaneously injected with artemisinin (25 mg/kg) 4 days after infection with the *P. yoelii* 17XNL parasite. Blood samples were collected before and after drug treatment and analysed using the XN-30 system (see also Fig. 5a).

### Statistical analyses

The correlation between parasitaemia determined using the XN-30 system and by microscopy was analysed using regression analysis. The coefficient of determination (R^2^) was calculated using Microsoft Excel (Microsoft, Redmond, WA, USA). Mean, standard deviation (SD), and coefficient of variation (CV % = SD/mean × 100) were calculated using Microsoft Excel (Microsoft) and Graphpad Prism version 5.0 (Graphpad Software, San Diego, CA, USA). The statistical significance of differences between non-infected and infected groups was evaluated through one-way analysis of variance followed by Dunnett’s multiple comparison tests using Graphpad Prism version 5.0 (Graphpad Software).

## Results

### Evaluation of blood samples from non-infected healthy mice using the XN-30 analyzer

**Fig. 1:**
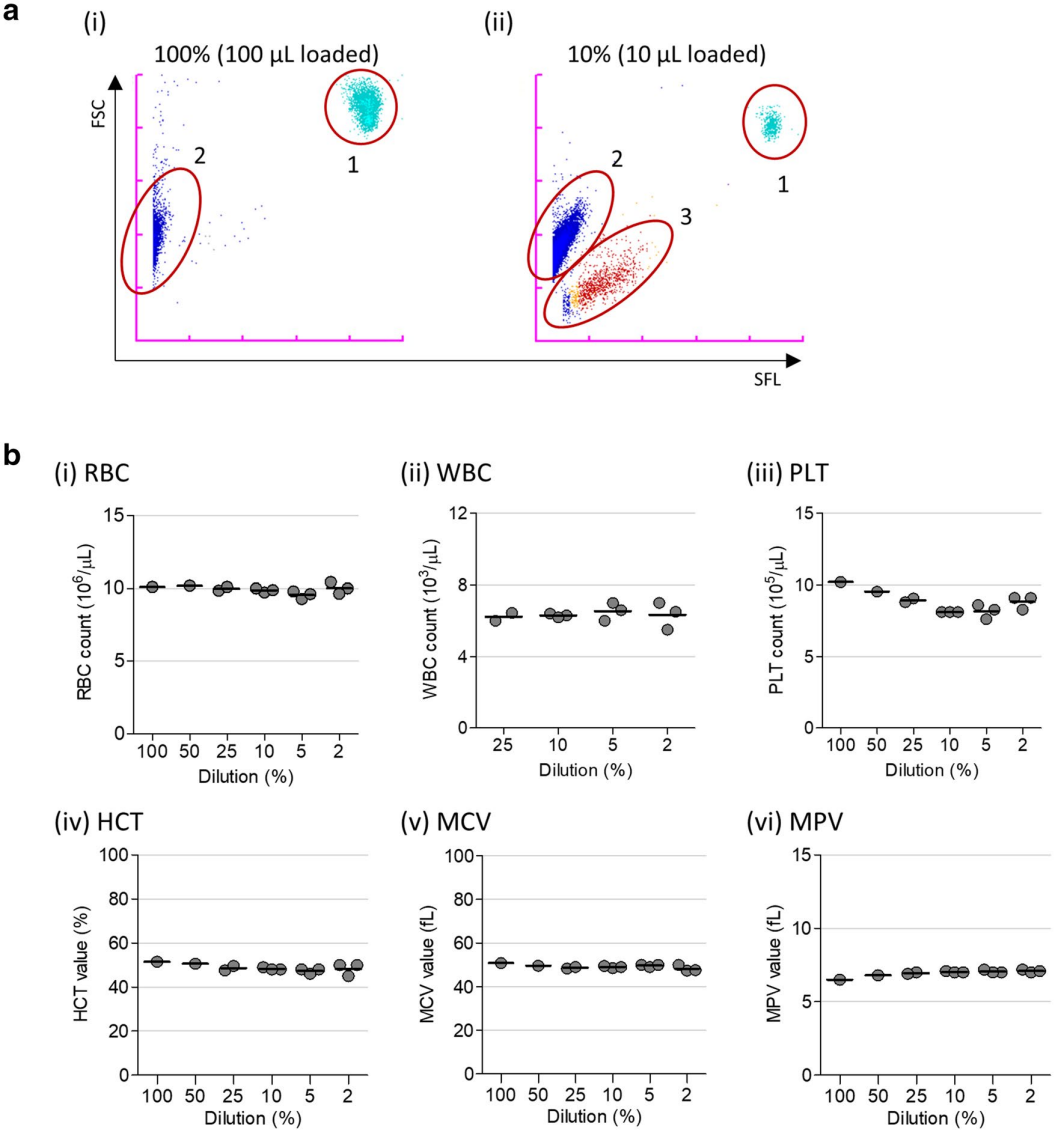
XN-30 analyzer data of blood samples from healthy humans and non-infected mice. **a** M scattergrams obtained from non-diluted human blood samples (i) and tenfold diluted mouse blood sample (ii). The ‘1’, ‘2’, and ‘3’ indicate white blood cells (WBCs), polychromatic red blood cells (RBCs), and Howell–Jolly body-containing RBCs (HJB-RBCs), respectively. Blue, polychromatic RBCs; light blue, WBCs; red, ring-forms; orange, trophozoites; purple, schizont were assigned based on default setting of the XN-30 analyzer; however, these were misclassified for mouse blood sample. **b** Dot-plot of RBC (i), WBC (ii), and PLT (iii) counts, and HCT (iv), MCV (v), and MPV (vi) values in blood samples from female mice. Horizontal bars represent means. RBC, WBC, and PLT counts, as well as HCT value were calculated according to the indicated dilution ratio. Data of the blood samples from male mice are shown in Additional file 1: Figure S1

As a first step to apply the XN-30 analyzer for the detection and measurement of iRBCs in mouse, blood samples from non-infected healthy mice were measured. Signals from non-diluted blood samples were saturated (100%, Additional file 1: Figure S1a, b); however, the blood samples diluted 10 to 50-fold were adequately represented on the M scattergram (female, Additional file 1: Figure S1a; male, Additional file 1: Figure S1b). For healthy human peripheral blood samples, the XN-30 analyzer distinguished WBCs (1) and polychromatic RBCs (2) on the M scattergram (Fig. 1a(i)); however, HJB-RBCs (3) were also detected for the mouse peripheral blood samples. Unfortunately, HJB-RBCs were recognized as ring-forms, trophozoites, and polychromatic RBCs (Fig. 1a(ii) and Additional file 1: Figure S1a). These findings suggest that the optimal working dilution for the samples needs to be determined and that HJB-RBCs hindered the precise enumeration of iRBCs. Moreover, as the XN-30 analyzer simultaneously reports other parameters such as RBC, WBC, and PLT counts, as well as HCT, MCV, and MPV values [7], the reliability of the data from diluted blood samples was also evaluated by comparing the counts and values of these parameters across varying dilutions. The comparison indicated that the dilutions hardly affected the haematology results (female, Fig. 1b; male, Additional file 1: Figure S1c). From these data, it can be inferred that 2 μL (1:50 dilution) of blood sample is sufficient for suitable measurements.

### Initial evaluation and re-analysis of the scattergrams obtained from *P. yoelii*-infected blood samples

**Fig. 2:**
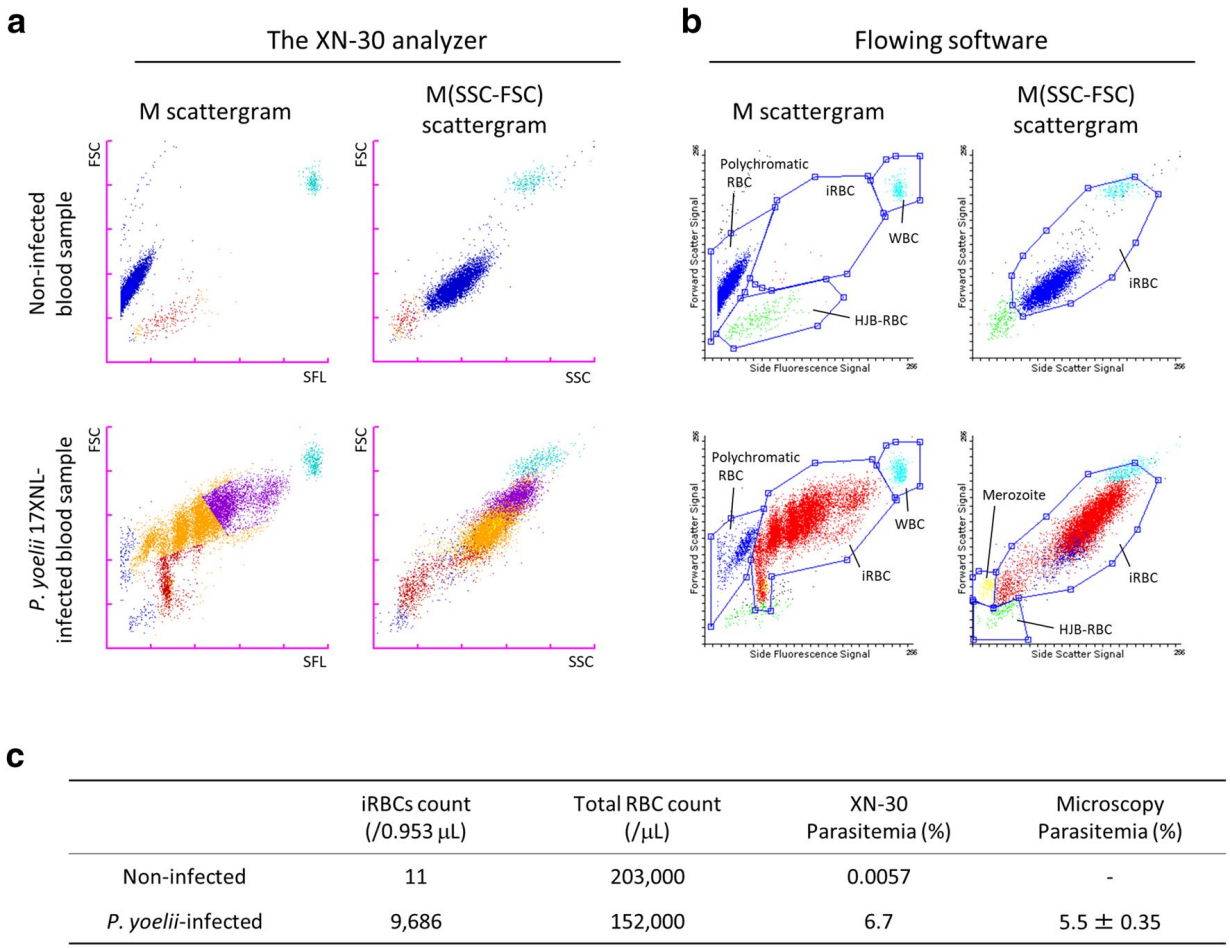
Re-analysis of the scattergrams by using the Flowing software. **a** The M and M(SSC-FSC) scattergrams measured by the XN-30 analyzer. (Upper panels) non-infected blood sample. (Lower panel) infected blood sample. Red, ring-forms; orange, trophozoites; purple, schizont were assigned based on the default setting of the XN-30 analyzer; however, these were misclassified. **b** The M and M(SSC-FSC) scattergrams re-analysed using the Flowing software. (Upper panels) non-infected blood sample. (Lower panels) infected blood sample. The iRBCs, WBCs, polychromatic RBCs, HJB-RBCs, and merozoites were gated. Red, iRBCs; blue, polychromatic RBCs; green, HJB-RBCs; yellow, merozoites; light blue, WBCs; black, uncharacterized cells. The scattergrams were obtained from blood samples diluted at 1:50. **c** Table of iRBC and total RBC counts and their parasitaemias. The dots per 0.953 μL of sample are shown on the scattergrams

The XN-30 analyzer showed WBCs, polychromatic RBCs, and HJB-RBCs in non-infected mouse blood samples on the M scattergram (Fig. 2a, upper left panel), similar to that in Fig. 1a(ii). The M(SSC-FSC) scattergram using the SSC parameter instead of the SFL parameter revealed the separation of these cells (Fig. 2a, upper right panel). However, HJB-RBCs were still misrecognized as ring-form parasites (Fig. 2a, upper panels). In infected blood samples, the analyzer detected iRBCs and HJB-RBCs, but was not able to distinguish the different developmental stages of the parasite (especially the trophozoite and schizont stages), owing to high multiple infection (Fig. 2a, lower panels and Additional file 1: Figure S4). Moreover, polychromatic RBCs and HJB-RBCs were again misrecognized as trophozoite and ring-forms. Thus, the data obtained from the analyzer require further analysis to resolve these inherent limitations. To overcome these issues, the scattergrams were analysed using the Flowing software in the XN-30 system described in the “Methods”.

Manual gating distinguished polychromatic RBCs, HJB-RBCs, and WBCs from iRBCs on the M scattergram; however, any dots in the area of the iRBCs were counted as iRBCs on the M scattergram, even in non-infected blood samples (Fig. 2b, upper panels). For infected blood samples, merozoites can be resolved in addition to HJB-RBCs on the M(SSC-FSC) scattergram (Fig. 2b, lower panels). Common dots in the iRBC areas on both the M and M(SSC-FSC) scattergrams were assigned as iRBCs. This analysis showed that the iRBC counts were 11 and 9686 (parasitaemias were 0.0057% and 6.7%, respectively) in the non-infected and infected blood samples, respectively (Fig. 2c); with 0.0057% of the parasitaemia in the non-infected blood sample regarded as false-positive. Further analysis of non-infected blood samples from female and male mice revealed false-positive parasitaemia values of 0.0043 ± 0.0013% and 0.011 ± 0.0057%, respectively (Additional file 2: Table S1). Of note, for infected blood samples, the 6.7% parasitaemia from the XN-30 system was similar to the 5.5 ± 0.35% parasitaemia determined through microscopy (Fig. 2c, lower line). These results indicate that the three-dimensional analysis using the SSC parameter added to the FSC and SFL parameters (see “Data analysis by Flowing software” in “Methods”) permitted a more accurate determination of iRBC count in mouse blood samples.

### Parasitaemia and haematological evaluation of the blood samples from infected mice by using the XN-30 system

**Fig. 3:**
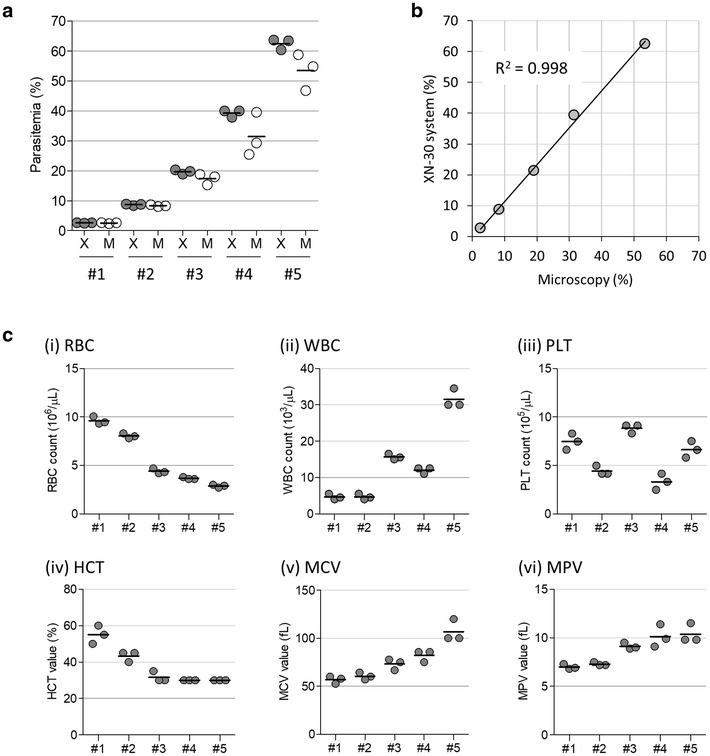
Comparison between parasitaemias determined using the XN-30 system and microscopy. **a** Dot-plot of parasitaemias determined using the XN-30 system and microscopy. Data from 5 types of blood samples are compared. Grey and white dots indicate parasitaemias obtained using the XN-30 system (denoted as ‘X’) and microscopy (denoted as ‘M’), respectively. Horizontal bars represent means calculated with values from three individual measurements. All data are provided in Additional file 1: Figure S2 and Additional file 2: Table S2. **b** Scatterplot of parasitaemias obtained from the XN-30 system and microscopy. Data are summarized with mean values from each sample shown in (**a**). R^2^ indicates the coefficient of determination. The diagonal line represents the regression line. **c** Dot-plot of RBC (i), WBC (ii) and PLT (iii) counts, and HCT (iv), MCV (v) and MPV (vi) values. Horizontal bars represent means. Data were obtained from blood samples diluted at 1:50

To further test the reliability of the XN-30 system, 5 mouse blood samples with different parasitaemias were compared using the XN-30 system and microscopy. The XN-30 system had less dispersion in a single sample (CV% = 2.36–2.96 in the XN-30 system; 3.14–18.93 in microscopy) and larger mean parasitaemia (XN-30/microscopy = 1.02–1.25) than that observed through microscopy (Fig. 3a, Additional file 1: Figure S2 and Additional file 2: Table S2). However, correlation analysis revealed a high coefficient of determination (R^2^ = 0.998) between the two (Fig. 3b). With these results, XN-30 system is potentially more reproducible and sensitive than microscopy. Furthermore, in addition to the parasitaemia, it was observed that RBC, WBC, and PLT counts, as well as HCT, MCV, and MPV values were reliable even using diluted samples (see Fig. 1b). When infected, with parasitaemia, haematological parameters fluctuated as expected (Fig. 3c, also see below).

**Fig. 4:**
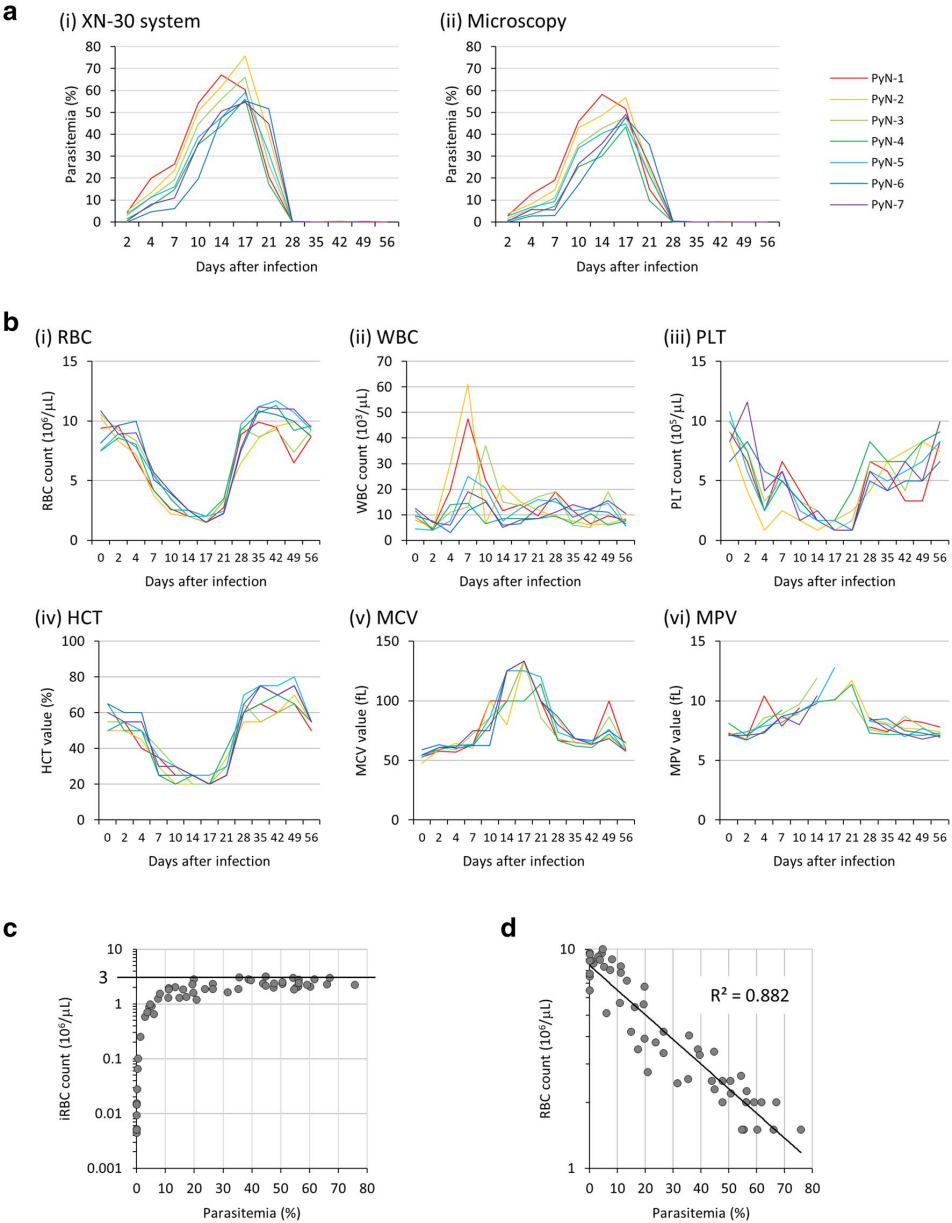
Sequential analysis of parasitaemia, RBC, WBC, and PLT counts, and HCT, MCV, and MPV values after infection. **a** Parasitaemias measured using the XN-30 system (i) and through microscopy (ii). All data are provided in Additional file 2: Table S3. **b** RBC (i), WBC (ii), and PLT (iii) counts, and HCT (iv), MCV (v), and MPV (vi) values. No values in MPV (vi) are out of range of the XN-30 analyzer (see also Additional file 2: Table S9). Horizontal bars represent means. These figures were calculated according to the dilution ratio (1:50). All data are provided in Additional file 2: Table S4 (RBC), S5 (WBC), S6 (PLT), S7 (HCT), S8 (MCV), and S9 (MPV). **c** Scatter-plot of iRBC counts and parasitaemia. Horizontal bar indicates 3 × 10^6^ iRBCs/μL. **d** Scatter-plot of RBC count and parasitaemia. R^2^ indicates the coefficient of determination. Data were obtained from blood samples diluted at 1:50

The course of infection was monitored using both the XN-30 system and microscopy. Both methods showed increase in parasitaemia until day 14–17 after which parasitaemia declined (Fig. 4a and Additional file 2: Table S3). A higher mean parasitaemia was obtained with the XN-30 system than with microscopy (Fig. 4a and Additional file 2: Table S3), similar to the results presented in Fig. 3a and Additional file 2: Table S2. A correlation analysis indicated a strong coefficient of determination (R^2^ = 0.973) (Additional file 1: Figure S3), as mentioned above (see Fig. 3b). Likewise, in addition to parasitaemia, the XN-30 analyzer was able to evaluate other parameters. RBC count and HCT value decreased as parasitaemia increased, and both values returned to baseline as parasitaemia is cleared (Fig. 4b(i), (iv)). This fluctuation matches with the anaemia status of mice after infection. However, WBC fluctuation differed largely in individual mice (Fig. 4b(ii)), rising rapidly after infection and decreasing around 14 days when parasitaemia is at its peak. The PLT count decreased as parasitaemia increased; however, its return to baseline was delayed compared with that of the RBC count and HCT value (Fig. 4b(iii)). Fluctuations in MCV and MPV values followed those of parasitaemia (Fig. 4b(v), (vi)). An increase in MCV and MPV values corresponded with the enlarged morphologies observed by microscopy (Additional file 1: Figure S4). The comparison of the iRBC count (iRBCs/μL) with parasitaemia (%) revealed that iRBC levels plateaued at approximately 3.0 × 10^6^ iRBCs/μL when the parasitaemia was approximately more than 20% (Fig. 4c). This finding indicates that the increase in parasitaemia for *P. yoelii* 17XNL was dependent on reductions in the levels of total RBCs greater than 20% parasitaemia. To validate this finding, RBC counts were correlated with parasitaemia. The comparison revealed a logarithmic negative correlation (Fig. 4d), suggesting that the total RBC levels were logarithmically reduced after infection and that the maximum concentration of iRBCs is regulated by uncharacterized mechanisms in mice and/or parasites.

### Application of the XN-30 system to evaluate the efficacy of anti-malarial drugs

**Fig. 5:**
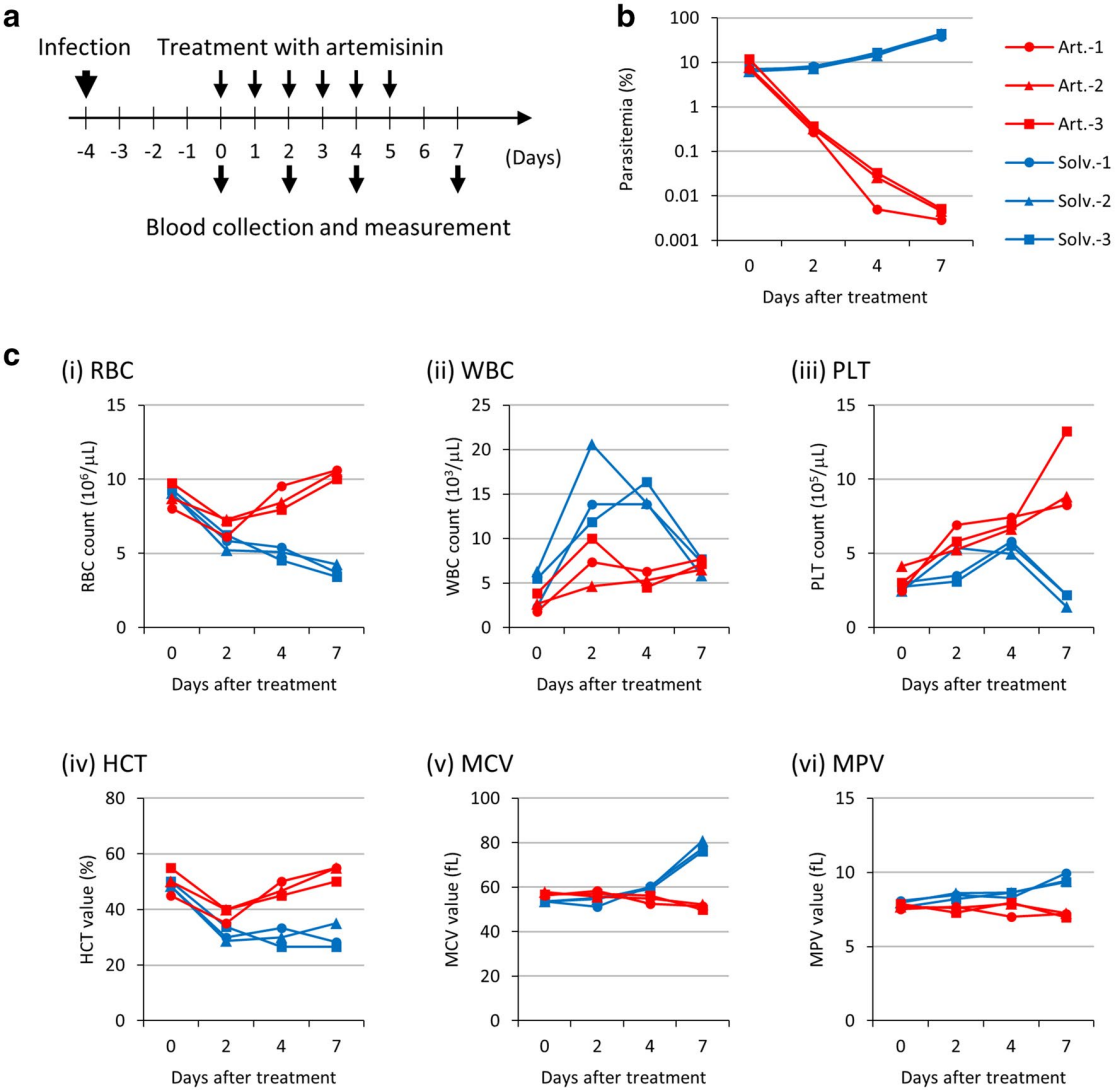
Analysis of parasitaemias, RBC, WBC, and PLT counts, and HCT, MCV, and MPV values after treatment with artemisinin **a** Schedule of infection, treatment, and measurement. **b** Parasitaemias measured using the XN-30 system. Red and blue lines indicate artemisinin- and solvent-treated mice, respectively. All data are provided in Additional file 1: Figure S5 and Additional file 2: Table S10. **c** RBC (i), WBC (ii), and PLT (iii) counts, and HCT (iv), MCV (v), and MPV (vi) values. Data were obtained from blood samples diluted at 1:50

To evaluate the efficacy of anti-malarial drugs with the XN-30 system, a rodent model for malaria was used to analyse parasite-infected mouse blood before and after artemisinin treatment. Mice were infected with the parasite on day-4 and then treated with artemisinin or solvent (Fig. 5a). Parasitaemia promptly decreased after artemisinin treatment (Fig. 5b; red lines) while it increased in solvent-treated mice (Fig. 5b; blue lines). Haematological parameters fluctuated in both artemisinin- and non-treatment groups. With artemisinin, treatment resulted in the recovery of RBC, PLT counts and HCT value, while WBC count, MCV, and MPV values barely fluctuated from baseline (Fig. 5c). From these results, XN-30 analyzer permits, aside from parasitaemia determination, the haematological evaluation in vivo and provide clues as to the curative effects of anti-malarial drugs.

## Discussion

The XN-30 analyzer was previously reported to allow the automatic detection of iRBCs in human blood samples and in vitro cultures [7, 8]. However, the default setting of the XN-30 analyzer is insufficient for the precise detection of iRBCs from mouse blood sample. This is further substantiated by analysis of blood samples from non-infected healthy mice. The third parameter of the SSC channel was tapped for another approach using three-dimensional analysis adding SSC parameter to FSC and SFL parameters.

Although the analysis of FCS files using Flowing software allowed for more precise iRBC counts in infected blood samples, false-positive dots were counted as iRBCs even in non-infected blood samples (Fig. 2 and Additional file 2: Table S1). This recognition was 0.0043 ± 0.0013% and 0.011 ± 0.0057% in non-infected female and male mice, respectively (Additional file 2: Table S1). The baseline level of false-positive values became higher after clearance of the parasites compared to naïve blood samples as RBCs containing dead parasites, HJB-RBCs, and polychromatic RBCs are being counted as iRBC. The contribution of gametocytes to the parasitaemia count was regarded as low, since gametocytes were not found under the microscope. However, further studies to evaluate the influence of gametocytes in the parasitaemia count is indeed necessary. Based on the present study, less than 0.02% parasitaemia was considered false-positive (Fig. 4a and Additional file 2: Table S3). This value is higher than that obtained with in vitro *P. falciparum* culture and human blood samples (~ 0.0004%, calculated from 18 iRBCs/μL) [7].

The XN-30 system detected HJB-RBCs that are generally present in healthy mouse blood (Fig. 1(ii)). The XN-30 system determined that the frequencies of HJB-RBCs were 0.12 ± 0.011% (female) and 0.21 ± 0.026% (male) (Additional file 2: Table S1). These values were nearly comparable with previously reported values of 0.16% [9], 0.2 ± 0.2% [10], 0.3% [11], and 0.52% [5] in mouse blood samples. Healthy human blood samples hardly contain HJB-RBCs; and when found in humans, HJB-RBCs is associated with abnormal splenic functions including asplenia or post-splenectomy [12]. Autosplenectomy resulting from sickle cell disease caused by a mutation for malaria resistance induces important clinical consequences [13]. Other causes are radiation therapy involving the spleen, such as that used to treat Hodgkin lymphoma [14]. HJB-RBCs are also observed in amyloidosis [15], severe haemolytic anaemia such as thalassaemia [16], megaloblastic anaemia [17], hereditary spherocytosis [18], heterotaxy with asplenia [19], and myelodysplastic syndrome [20]. Thus, the quantitative measurement capability of the XN-30 system for HJB-RBCs would be useful for clinical evaluation.

The XN-30 analyzer detected polychromatic RBCs and revealed fluctuation in their levels during infection. This fluctuation reflects erythropoietic activity [21]. Although the XN-30 analyzer fails to detect polychromatic RBCs with lower signal intensity than the cut-off value in the current setting of the SFL channel, at its present setting counting of polychromatic RBCs can be achieved and this technique would be useful for the evaluation of erythropoietic activity.

For more accurate calculation of parasitaemia, the iRBC region on the M and M(SSC-SFC) scattergrams was manually gated for every infected blood sample. Further consideration regarding an automated gating method will be required for high-throughput analysis of parasite-infected blood samples.

The present study focused on C57BL/6 mice infected with the *P. yoelii* 17XNL strain, but *P. yoelii* 17XL and *P. berghei* ANKA strains and ICR mice have also been examined. With any combination of mouse and parasite strain, similar results were obtained (Additional file 1: Figure S6). The XN-30 analyzer was capable of detecting parasitaemia in infected mice, as well as permitting the simultaneous analysis of haematological parameters. Based on this it is also inferred that the XN-30 analyzer should be useful for the detection of *Babesia* and *Bartonella*, which also infect RBCs [22].

## Conclusion

The XN-30 system permits simple, rapid, and precise measurement of iRBCs in mouse blood samples infected with rodent malarial parasites. Simultaneous haematological evaluation offers an important tool for correlating the physiological status of malaria-infected mice as well as offering reliable, unbiased automation. Together with the ability of the XN-30 analyzer to detect iRBCs in patient blood, conduct biological analyses of erythrocytic-stage parasites, this system is also now available for use in rodent models to study, screen and develop anti-malarial drugs.

## Additional files

**Additional file 1: Figure S1.** Analysis of data obtained using the XN-30 analyzer on blood samples from healthy non-infected mice, related to Fig. 1. **Figure S2.** M scattergrams re-analysed using the Flowing software after parasite infection. **Figure S3.** Comparison between parasitaemias determined using the XN-30 system and microscopy. **Figure S4.** Morphological evidence of the high MCV and MPV values in infected blood samples by microscopy. **Figure S5.** M scattergrams re-analysed using the Flowing software after treatment with artemisinin. **Figure S6.** Re-analysed scattergrams of mouse blood samples infected with parasites.

**Additional file 2: Table S1.** Data of false-positive iRBCs and HJB-RBCs in non-infected mice. **Table S2.** Comparison of parasitaemias obtained using the XN-30 system and microscopy, related to Fig. 3. **Table S3.** Comparison of parasitaemia, related to Fig. 4a. **Table S4.** Sequential analysis of RBC count, related to Fig. 4b(i). **Table S5.** Sequential analysis of WBC count, related to Fig. 4b(ii). **Table S6.** Sequential analysis of PLT value count, related to Fig. 4b(iii). **Table S7.** Sequential analysis of HCT value, related to Fig. 4b(iv). **Table S8.** Sequential analysis of MCV value, related to Fig. 4b(v). **Table S9.** Sequential analysis of MPV value, related to Fig. 4b(vi). **Table S10.** Sequential analysis of parasitaemia and the parameters after treatment with artemisinin, related to Fig. 5.

## Abbreviations

RBC: red blood cell
iRBC: infected RBC
HJB-RBC: Howell–Jolly body-containing RBC
WBC: white blood cell
PLT: platelet
HCT: haematocrit
MCV: mean corpuscular volume
MPV: mean platelet volume
FSC: forward scattered light
SFL: side fluorescent light
SSC: side scattered light
PBS: phosphate-buffered saline
R^2^: coefficient of determination
SD: standard deviation
CV: coefficient of variation
RIMD: Research Institute for Microbial Diseases
