# Supplementary material for: Application of the automated haematology analyzer XN-30 in an experimental rodent model of malaria
Source: Malar J. 2018 Apr 16;17:165. doi: 10.1186/s12936-018-2313-6 (PMC5902832; doi:10.1186/s12936-018-2313-6)
Supplement: Supplementary file 1 — Additional file 1: Figure S1. Analysis of data obtained using the XN-30 analyzer on blood samples from healthy non-infected mice, related to Fig. 1. Figure S2. M scattergrams re-analysed using the Flowing software after parasite infection. Figure S3. Comparison between parasitaemias determined using the XN-30 system and microscopy. Figure S4. Morphological evidence of the high MCV and MPV values in infected blood samples by microscopy. Figure S5. M scattergrams re-analysed using the Flowing software after treatment with artemisinin. Figure S6. Re-analysed scattergrams of mouse blood samples infected with parasites. [file 12936_2018_2313_MOESM1_ESM.pdf]

Fig. S1

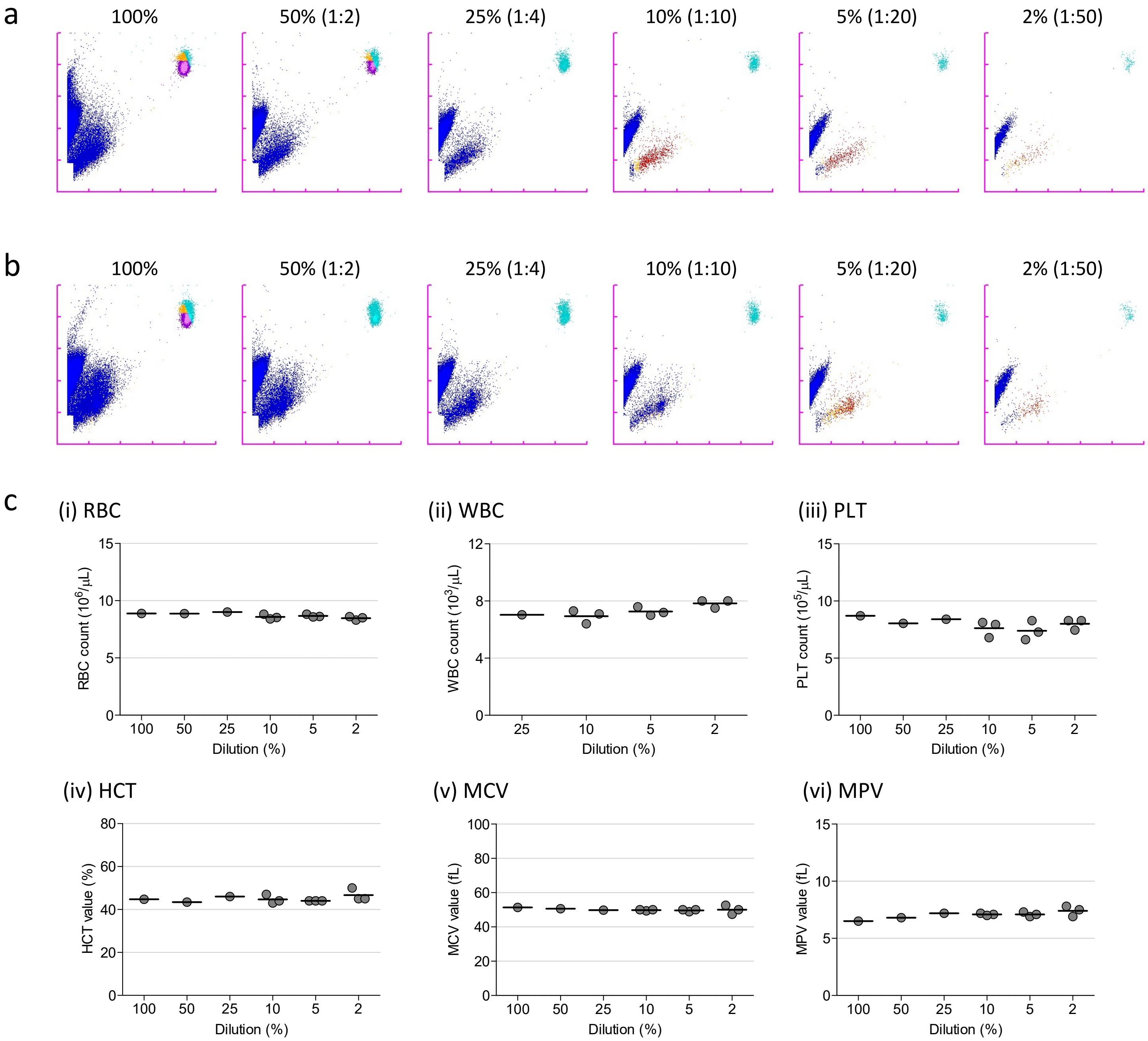

a
100%
50% (1:2)
25% (1:4)
10% (1:10)
5% (1:20)
2% (1:50)
b
100%
50% (1:2)
25% (1:4)
10% (1:10)
5% (1:20)
2% (1:50)
c
(i) RBC
RBC count (10^6/μL)
Dilution (%)
(ii) WBC
WBC count (10^3/μL)
Dilution (%)
(iii) PLT
PLT count (10^5/μL)
Dilution (%)
(iv) HCT
HCT value (%)
Dilution (%)
(v) MCV
MCV value (fL)
Dilution (%)
(vi) MPV
MPV value (fL)
Dilution (%)

Fig. S1: Analysis of data obtained using the XN-30 analyzer on blood samples from healthy non-infected mice, related to Fig. 1 (**a** and **b**) M scattergrams of mouse blood samples with different dilution rate. (**a**) Female. (**b**) Male. Blood samples (1- to 50-fold dilution) were analysed using the XN-30 analyzer. Red, ring-forms; orange, trophozoites; purple, schizont were assigned in default setting of XN-30 analyzer; however, these were misrecognized. (**c**) Dot-plot of values of RBC (i), WBC (ii), and PLT (iii) counts, and HCT (iv), MCV (v), and MPV (vi) values of male mouse blood samples. Horizontal bars represent the means. These figures were calculated according to the indicated dilution ratio. Data of the female mouse blood sample are shown in Fig. 1.

Fig. S2

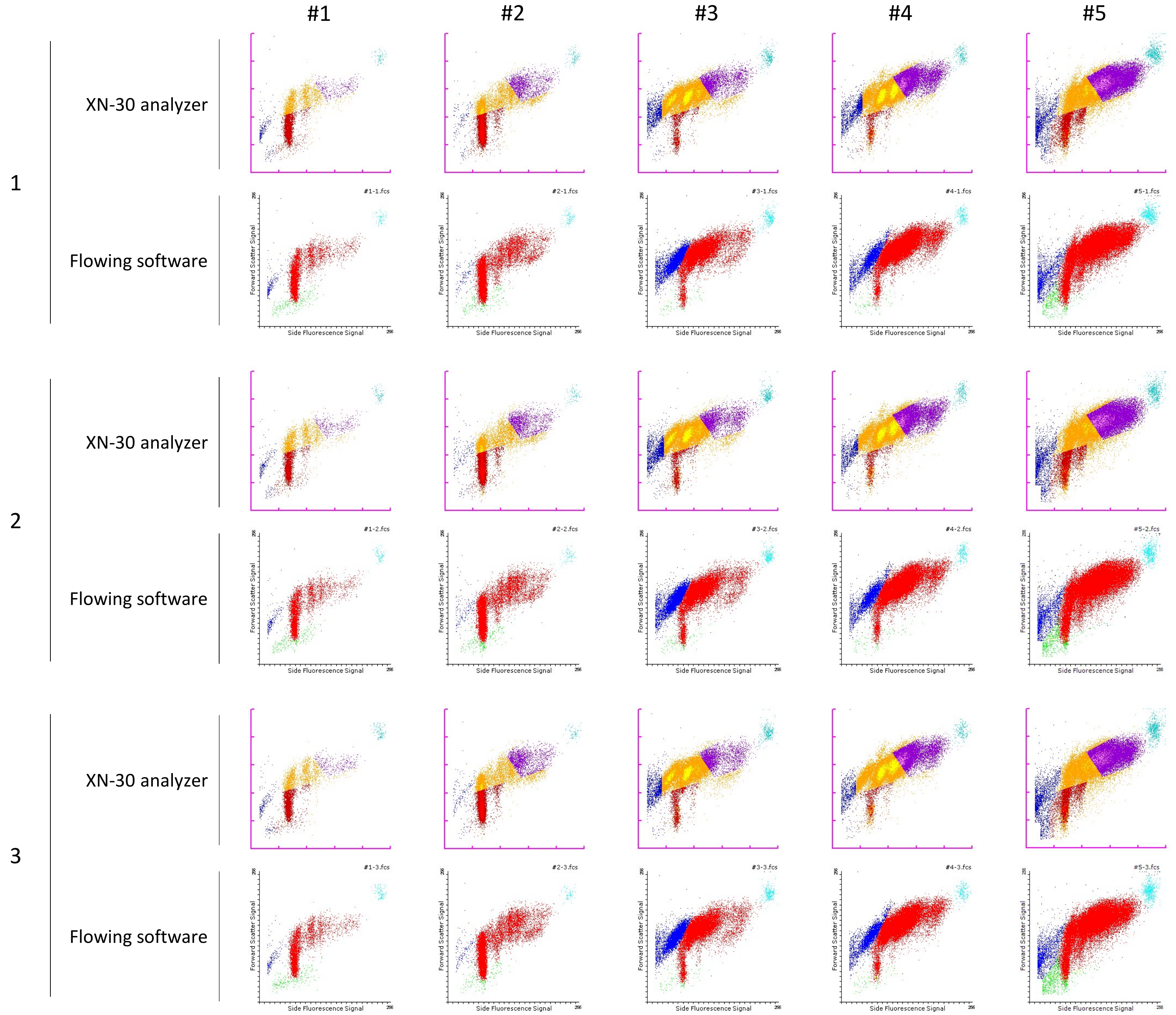

#1
#2
#3
#4
#5
1
2
3
XN-30 analyzer
Flowing software
Forward Scatter Signal
Side Fluorescence Signal
#1-1.fcs
#2-1.fcs
#3-1.fcs
#4-1.fcs
#5-1.fcs
#1-2.fcs
#2-2.fcs
#3-2.fcs
#4-2.fcs
#5-2.fcs
#1-3.fcs
#2-3.fcs
#3-3.fcs
#4-3.fcs
#5-3.fcs

Fig. S2: M scattergrams re-analysed using the Flowing software after parasite infection The M scattergrams that are original (upper panels) and re-analysed using the Flowing software (lower panels). (Upper panels) Red, ring-forms; orange, trophozoites; purple, schizont were assigned in the default setting of the XN-30 analyzer; however, these were misrecognized. (Lower panels) Red, iRBCs; blue, polychromatic RBCs; green, HJB-RBCs; yellow, merozoites; light blue, WBCs; and black, uncharacterized cells. These scattergrams were obtained from blood samples diluted at 1:50.

Fig. S3

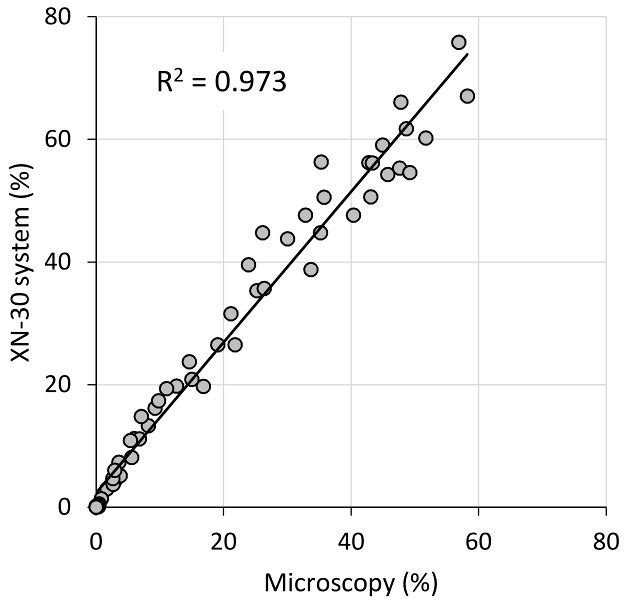

Fig. S3: Comparison between parasitemias determined using the XN-30 system and by microscopy. R^2 indicates the coefficient of determination. The diagonal line represents the regression line.

Fig. S4

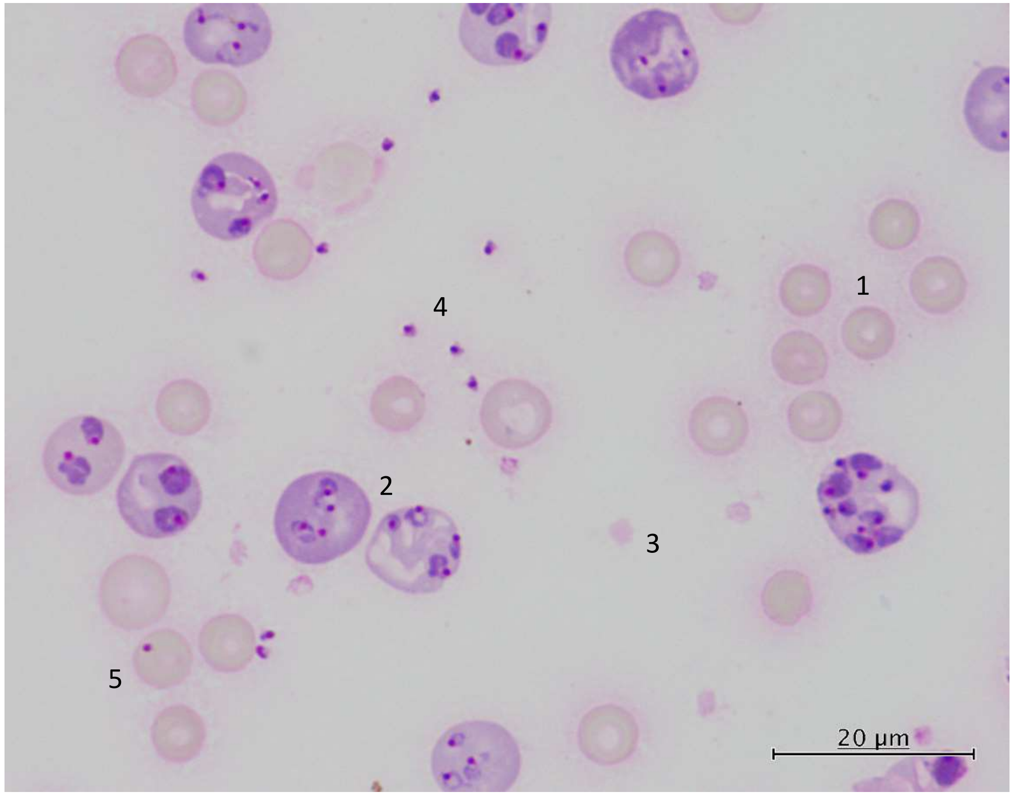

Fig. S4: Morphological evidence of the high MCV and MPV values in infected blood samples by microscopy 1, RBC; 2, iRBC; 3, platelet; 4, merozoite; and 5, HJB-RBC. The scale bar represents 20 μm.

Fig. S5

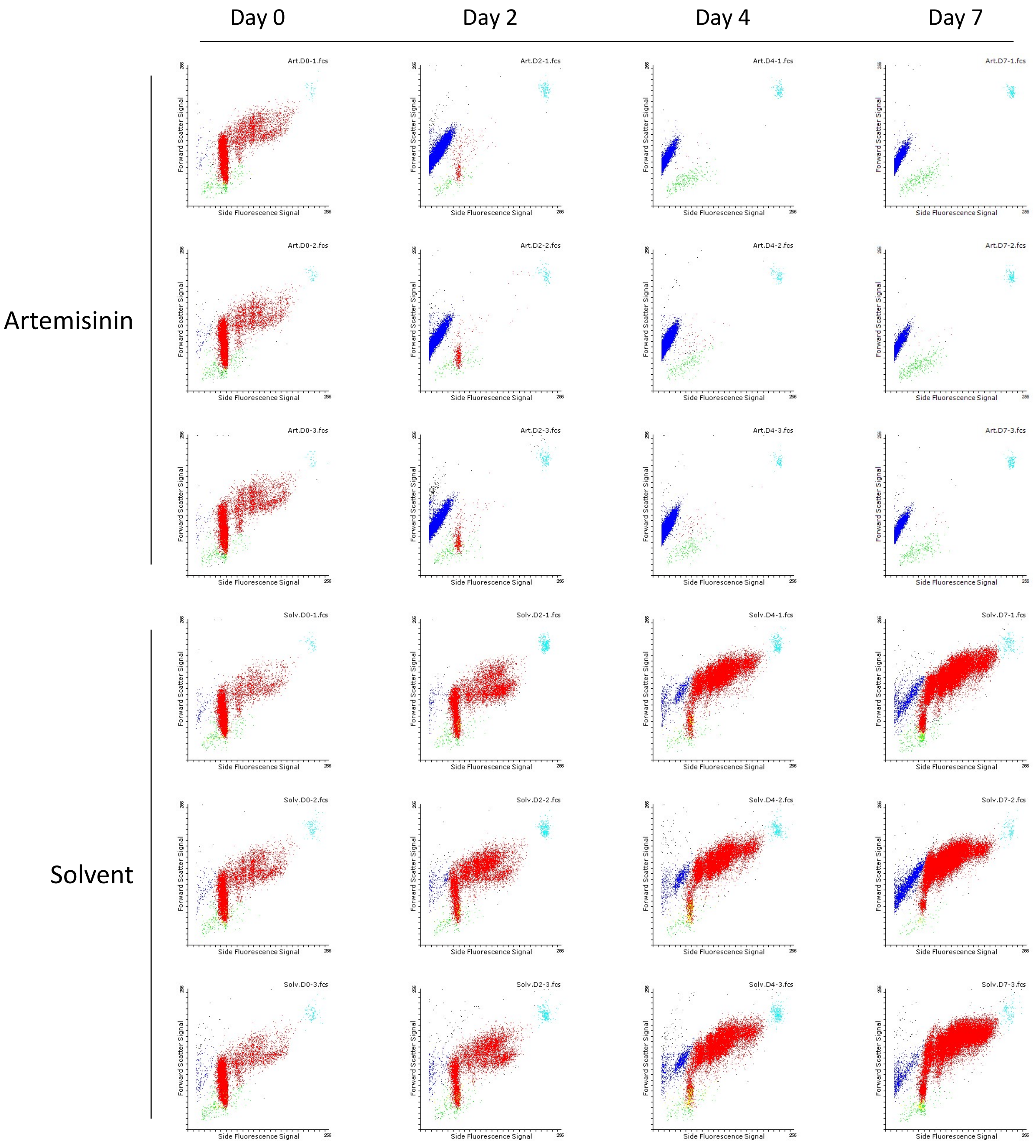
Day 0
Day 2
Day 4
Day 7
Artemisinin
Solvent
Forward Scatter Signal
Side Fluorescence Signal
Art.D0-1.fcs
Art.D2-1.fcs
Art.D4-1.fcs
Art.D7-1.fcs
Art.D0-2.fcs
Art.D2-2.fcs
Art.D4-2.fcs
Art.D7-2.fcs
Art.D0-3.fcs
Art.D2-3.fcs
Art.D4-3.fcs
Art.D7-3.fcs
Solv.D0-1.fcs
Solv.D2-1.fcs
Solv.D4-1.fcs
Solv.D7-1.fcs
Solv.D0-2.fcs
Solv.D2-2.fcs
Solv.D4-2.fcs
Solv.D7-2.fcs
Solv.D0-3.fcs
Solv.D2-3.fcs
Solv.D4-3.fcs
Solv.D7-3.fcs

Fig. S5: M scattergrams re-analysed using the Flowing software after treatment with artemisinin Red, iRBCs; blue, polychromatic RBCs; green, HJB-RBCs; yellow, merozoites; light blue, WBCs; and black, uncharacterized cells. These scattergrams were obtained from blood samples diluted at 1:50.

Fig. S6

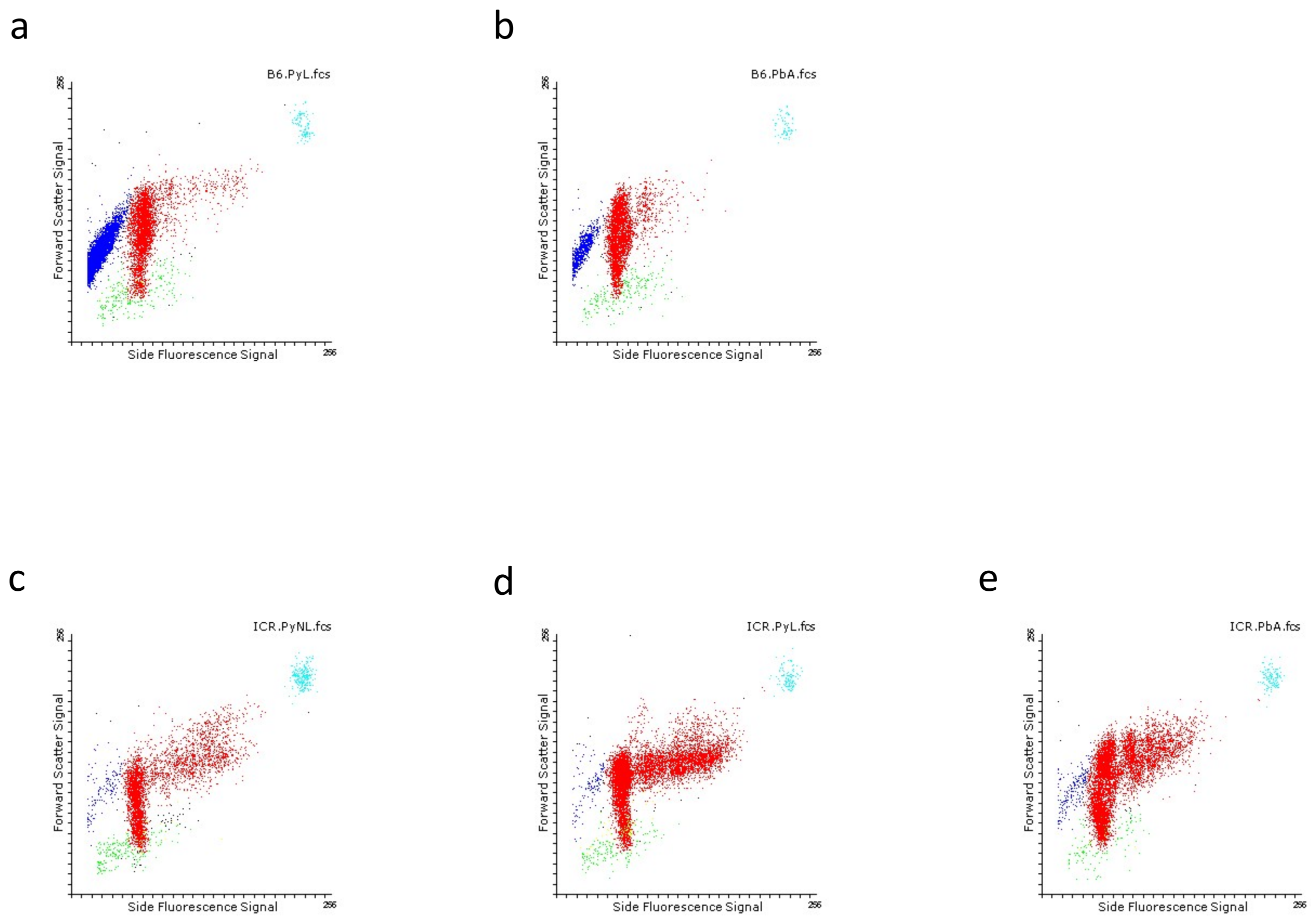
a
B6.PyL.fcs
Forward Scatter Signal
Side Fluorescence Signal
256
b
B6.PbA.fcs
Forward Scatter Signal
Side Fluorescence Signal
256
c
ICR.PyNL.fcs
Forward Scatter Signal
Side Fluorescence Signal
256
d
ICR.PyL.fcs
Forward Scatter Signal
Side Fluorescence Signal
256
e
ICR.PbA.fcs
Forward Scatter Signal
Side Fluorescence Signal
256

Fig. S6: Re-analysed scattergrams of mouse blood samples infected with parasites

The M scattergrams re-analysed using the Flowing software. (**a**) Blood samples of C57BL/6 mice infected with the *P. yoelii* 17XL strain. (**b**) Blood samples of C57BL/6 mice infected with *P. berghei* ANKA strain. (**c**) Blood samples of ICR mice infected with *P. berghei* ANKA strain. (**d**) Blood samples of ICR mice infected with *P. yoelii* 17XNL strain. (**e**) Blood samples of ICR mice infected with *P. yoelii* 17XL strain. Red, iRBCs; blue, polychromatic RBCs; green, HJB-RBCs; yellow, merozoites; light blue, WBCs; and black, uncharacterized cells. These scattergrams were obtained from blood samples diluted at 1:50.
