# Supplementary material for: Application of the automated haematology analyzer XN-30 in an experimental rodent model of malaria
Source: Malar J. 2018 Apr 16;17:165. doi: 10.1186/s12936-018-2313-6 (PMC5902832; doi:10.1186/s12936-018-2313-6)
Supplement: Supplementary file 2 — Additional file 2: Table S1. Data of false-positive iRBCs and HJB-RBCs in non-infected mice. Table S2. Comparison of parasitaemias obtained using the XN-30 system and microscopy, related to Fig. 3. Table S3. Comparison of parasitaemia, related to Fig. 4a. Table S4. Sequential analysis of RBC count, related to Fig. 4b(i). Table S5. Sequential analysis of WBC count, related to Fig. 4b(ii). Table S6. Sequential analysis of PLT value count, related to Fig. 4b(iii). Table S7. Sequential analysis of HCT value, related to Fig. 4b(iv). Table S8. Sequential analysis of MCV value, related to Fig. 4b(v). Table S9. Sequential analysis of MPV value, related to Fig. 4b(vi). Table S10. Sequential analysis of parasitaemia and the parameters after treatment with artemisinin, related to Fig. 5. [file 12936_2018_2313_MOESM2_ESM.pdf]

Table S1: Data of false-positive iRBCs and HJB-RBCs in non-infected mice

	Total RBC count (10^6/μL)*	iRBC count (/0.953 μL)*	iRBC (%)	iRBC Mean ± SD (%)	HJB-RBC count (/0.953 μL)*	HJB-RBC (%)	HJB-RBC Mean ± SD (%)
Female	0.203	11	0.0057	0.0043 ± 0.0013	220	0.11	0.12 ± 0.011
	0.188	8	0.0045		220	0.12	
	0.206	9	0.0046		195	0.10	
	0.211	7	0.0035		214	0.11	
	0.217	9	0.0044		234	0.11	
	0.201	9	0.0047		278	0.15	
	0.216	7	0.0034		260	0.13	
	0.220	5	0.0024		219	0.10	
	0.213	8	0.0039		237	0.12	
	0.211	4	0.0020		225	0.11	
	0.215	12	0.0059		231	0.11	
	0.195	7	0.0038		221	0.12	
	0.196	13	0.0070		239	0.13	
	0.200	5	0.0026		248	0.13	
	0.200	9	0.0047		198	0.10	
	0.209	9	0.0045		227	0.11	
	0.193	10	0.0054		219	0.12	
Male	0.241	21	0.0091	0.011 ± 0.0057	541	0.24	0.21 ± 0.026
	0.191	11	0.0060		326	0.18	
	0.224	19	0.0089		500	0.23	
	0.161	31	0.0202		378	0.25	
	0.177	20	0.0119		345	0.20	
	0.172	13	0.0079		333	0.20	
	0.181	12	0.0070		297	0.17	
	0.161	31	0.0202		376	0.25	
	0.175	19	0.0114		345	0.21	
	0.17	22	0.0136		343	0.21	
	0.164	24	0.0154		365	0.23	
	0.176	8	0.0048		301	0.18	
	0.161	36	0.0235		375	0.24	
	0.175	12	0.0072		340	0.20	
	0.184	10	0.0057		313	0.18	
	0.189	12	0.0067		319	0.18	
	0.192	9	0.0049		374	0.20	

* Values are from the blood samples diluted at 1:50.

Table S2: Comparison of parasitemias obtained using the XN-30 system and through microscopy, related to Fig. 3

	iRBCs count (/0.953 μL)*	Total RBC count (/μL)*	XN-30 system			Microscopy			XN-30/microscopy
			Parasitemia (%)**	mean ± SD	CV%	Parasitemia (%)	mean ± SD	CV%	(Total parasitemia)
	5,066	201,000	2.6			2.4			
#1	4,629	186,000	2.6	2.6 ± 0.06	2.36	2.7	2.5 ± 0.13	4.99	1.02
	4,506	189,000	2.5			2.6			
	12,253	154,000	8.3			8.7			
#2	12,186	144,000	8.9	8.7 ± 0.23	2.67	8.1	8.4 ± 0.26	3.14	1.04
	12,062	144,000	8.8			8.3			
	16,234	84,000	20.3			18.0			
#3	16,912	94,000	18.9	19.7 ± 0.58	2.96	15.3	17.4 ± 1.52	8.76	1.13
	16,226	86,000	19.8			18.8			
	27,065	71,000	40.0			39.6			
#4	27,074	75,000	37.9	39.3 ± 1.0	2.55	25.5	31.5 ± 5.96	18.93	1.25
	27,449	72,000	40.0			29.3			
	34,541	60,000	60.4			54.8			
#5	34,461	57,000	63.4	62.5 ± 1.5	2.39	46.8	53.5 ± 4.99	9.34	1.17
	32,776	54,000	63.7			58.8			

* Values are from the blood samples diluted at 1:50.

** Parasitemia (%) = iRBC count/0.953/total RBC count x 100

Table S3: Comparison of parasitemia, related to Fig. 4a

(i) XN-30 system

After infection	Parasitremia (%)											
	day 2	day 4	day 7	day 10	day 14	day 17	day 21	day 28	day 35	day 42	day 49	day 56
PyN-1	4.4	19.8	26.5	54.3	67.1	60.2	20.8	0.028	*0.0053*	0.022	0.20	*0.0049*
PyN-2	5.1	13.3	23.8	50.6	61.7	75.8	39.6	0.11	*0.0024*	*0.0044*	*0.0048*	*0.014*
PyN-3	3.0	11.2	19.3	44.8	56.2	66.1	26.5	*0.011*	*0.005*	0.33	0.033	*0.0054*
PyN-4	1.4	7.4	14.8	35.3	43.8	56.2	17.3	*0.0017*	*0.0023*	*0.0051*	*0.0074*	*0.0057*
PyN-5	3.7	11.2	16.2	38.8	47.6	59.1	31.6	*0.019*	*0.0038*	*0.0058*	*0.0034*	*0.0054*
PyN-6	0.046	4.7	6.1	19.7	47.7	55.3	51.6	0.098	*0.011*	*0.0045*	*0.0042*	*0.0034*
PyN-7	0.5	8.1	10.9	35.7	50.6	54.6	44.8	0.089	*0.018*	*0.0038*	*0.0019*	*0.0021*
Mean	2.6	10.8	16.8	39.9	53.5	61.0	33.2	0.051	0.0068	0.054	0.036	0.0058
SD	1.8	4.5	6.6	10.6	7.8	7.0	11.8	0.043	0.005	0.11	0.068	0.0035

(ii) Microscopy

After infection	Parasitremia (%)											
	day 2	day 4	day 7	day 10	day 14	day 17	day 21	day 28	day 35	day 42	day 49	day 56
PyN-1	3.0	12.6	19.1	45.7	58.2	51.7	15.0	0.021	nd	0.012	0.0085	0.0
PyN-2	3.8	8.2	14.6	43.1	48.7	56.9	23.9	0.092	nd	nd	nd	0.0
PyN-3	1.7	6.0	11.0	35.2	42.8	47.8	21.8	nd	nd	0.272	0.0042	0.0
PyN-4	0.8	3.6	7.1	25.2	30.0	43.3	9.8	nd	nd	nd	nd	0.0
PyN-5	2.7	6.8	9.2	33.7	40.4	44.9	21.1	nd	nd	nd	nd	0.0
PyN-6	0.0	2.6	2.9	16.8	32.8	47.6	35.3	0.023	nd	nd	nd	0.0
PyN-7	0.5	5.6	5.4	26.4	35.7	49.2	26.1	0.035	nd	nd	nd	0.0
Mean	1.8	6.5	9.9	32.3	41.2	48.8	21.9	-	-	-	-	-
SD	1.3	3.0	5.1	9.5	9.0	4.2	7.5	-	-	-	-	-

Italic values indicates less than 0.02% in (i) XN-30 system.

"nd", not determined in (ii) Microscopy.

Table S4: Sequential analysis of RBC count, related to Fig. 4b(i)

After infection	RBC count (x $10^6/\mu L$)												
	day 0	day 2	day 4	day 7	day 10	day 14	day 17	day 21	day 28	day 35	day 42	day 49	day 56
PyN-1	9.4	9.6	6.8	4.2	2.7	2.0	1.5	2.8	8.9	9.9	9.5	6.5	8.7
PyN-2	10.3	8.3	7.2	3.8	2.2	2.0	1.5	3.3	6.5	8.6	9.6	9.9	8.8
PyN-3	10.6	9.3	8.4	5.6	3.4	2.0	1.5	3.4	9.5	8.7	9.3	7.4	9.2
PyN-4	7.5	8.6	8.1	4.2	2.6	2.5	2.0	3.5	9.2	10.7	11.3	9.2	9.6
PyN-5	7.6	9.0	7.8	5.5	3.5	2.0	2.0	2.5	9.8	11.2	11.7	10.8	9.3
PyN-6	8.2	9.7	10.0	5.1	3.9	2.5	1.5	2.3	7.5	10.9	10.6	10.0	8.7
PyN-7	10.9	8.9	9.0	5.7	4.1	2.5	1.5	2.3	7.8	11.2	11.1	11.0	9.5
Mean	9.2	9.0	8.2	4.9***	3.2***	2.2***	1.6***	2.8***	8.4	10.2	10.4	9.3	9.1
SD	1.3	0.5	1.0	0.73	0.66	0.25	0.23	0.49	1.1	1.1	0.90	1.6	0.36

*** $p<0.001$

Table S5: Sequential analysis of WBC count, related to Fig. 4b(ii)

After infection	WBC count (x $10^3/\mu L$)												
	day 0	day 2	day 4	day 7	day 10	day 14	day 17	day 21	day 28	day 35	day 42	day 49	day 56
PyN-1	9.0	4.5	19.5	47.5	23.5	11.5	14.0	9.5	19.0	11.5	6.5	9.5	8.0
PyN-2	9.0	4.0	30.0	61.0	6.5	21.5	15.0	12.0	9.0	7.5	6.0	6.5	8.5
PyN-3	8.0	5.0	11.0	13.0	37.0	15.0	13.5	17.0	19.0	6.0	5.0	19.0	5.5
PyN-4	11.5	4.0	14.0	14.5	6.5	8.5	8.5	8.5	9.5	6.5	10.5	6.0	7.5
PyN-5	4.5	4.0	7.5	25.0	20.5	7.5	12.5	16.0	15.0	11.5	12.5	14.5	5.5
PyN-6	9.5	7.5	3.0	11.5	15.0	6.0	6.5	13.0	16.5	9.0	11.5	11.0	6.5
PyN-7	12.5	7.0	6.0	19.0	15.5	5.0	8.0	8.5	11.0	14.0	12.0	15.5	10.5
Mean	9.1	5.1	13.0	27.4***	17.8	10.7	11.1	12.1	14.1	9.4	9.1	11.7	7.4
SD	2.4	1.4	8.6	17.9	9.8	5.4	3.1	3.2	4.0	2.8	2.9	4.5	1.7

*** $p<0.001$

Table S6: Sequential analysis of PLT count, related to Fig. 4b(iii)

After infection	PLT count (x 10^5/μL)												
	day 0	day 2	day 4	day 7	day 10	day 14	day 17	day 21	day 28	day 35	day 42	day 49	day 56
PyN-1	9.1	6.6	2.5	6.6	4.1	1.7	1.7	0.8	6.6	5.8	3.3	3.3	8.3
PyN-2	8.3	4.1	0.8	2.5	1.7	0.8	1.7	2.5	4.1	6.6	7.5	8.3	7.5
PyN-3	9.1	7.5	3.3	5.0	3.3	2.5	0.8	1.7	6.6	6.6	4.1	8.3	9.1
PyN-4	9.9	7.5	2.5	5.0	3.3	1.7	1.7	4.1	8.3	6.6	6.6	8.3	9.1
PyN-5	10.8	5.8	2.5	5.8	2.5	1.7	1.7	0.8	5.8	5.0	5.8	6.6	8.3
PyN-6	6.6	8.3	5.8	5.0	3.3	1.7	0.8	0.8	5.0	4.1	5.0	5.0	6.6
PyN-7	8.3	11.6	4.1	5.8	1.7	2.5	0.8	0.8	5.8	4.1	6.6	5.0	9.9
Mean	8.9	7.3	3.1***	5.1***	2.8***	1.8***	1.3***	1.7***	6.0**	5.6***	5.6***	6.4*	8.4
SD	1.2	2.1	1.4	1.2	0.9	0.5	0.4	1.2	1.2	1.1	1.4	1.9	1.0

*, $p<0.05$; **, $p<0.01$, ***, $p<0.001$

Table S7: Sequential analysis of HCT value, related to Fig. 4b(iv)

After infection	HCT value (%)												
	day 0	day 2	day 4	day 7	day 10	day 14	day 17	day 21	day 28	day 35	day 42	day 49	day 56
PyN-1	50.0	55.0	40.0	35.0	25.0	25.0	20.0	25.0	60.0	65.0	60.0	65.0	50.0
PyN-2	50.0	50.0	45.0	30.0	20.0	20.0	20.0	35.0	55.0	55.0	60.0	70.0	55.0
PyN-3	55.0	55.0	50.0	40.0	30.0	20.0	20.0	30.0	65.0	55.0	60.0	65.0	55.0
PyN-4	65.0	50.0	50.0	25.0	20.0	25.0	20.0	40.0	60.0	65.0	70.0	65.0	55.0
PyN-5	50.0	55.0	50.0	35.0	30.0	25.0	25.0	30.0	70.0	75.0	75.0	80.0	55.0
PyN-6	65.0	60.0	60.0	25.0	25.0	25.0	20.0	25.0	65.0	75.0	70.0	75.0	55.0
PyN-7	60.0	55.0	55.0	30.0	30.0	25.0	20.0	25.0	60.0	75.0	70.0	75.0	55.0
Mean	56.4	54.3	50.0***	31.4***	25.7***	23.6***	20.7***	30.0***	62.1	66.4**	66.4**	70.7***	54.3
SD	6.4	3.2	6.0	5.2	4.2	2.3	1.7	5.3	4.5	8.3	5.8	5.6	1.7

**, p<0.01; ***, p<0.001

Table S8: Sequential analysis of MCV value, related to Fig. 4b(v)

After infection	MCV value (fL)												
	day 0	day 2	day 4	day 7	day 10	day 14	day 17	day 21	day 28	day 35	day 42	day 49	day 56
PyN-1	52.6	57.9	57.1	63.6	100.0	100.0	133.3	100.0	66.7	65.0	63.2	100.0	58.8
PyN-2	47.6	58.8	64.3	60.0	100.0	80.0	133.3	100.0	84.6	64.7	63.2	70.0	61.1
PyN-3	52.4	61.1	58.8	72.7	85.7	100.0	133.3	85.7	68.4	64.7	63.2	86.7	61.1
PyN-4	54.2	58.8	62.5	62.5	80.0	100.0	100.0	114.3	66.7	61.9	60.9	72.2	57.9
PyN-5	52.6	61.1	62.5	63.6	85.7	125.0	125.0	120.0	73.7	68.2	65.2	76.2	57.9
PyN-6	59.1	63.2	60.0	62.5	62.5	125.0	133.3	100.0	86.7	68.2	66.7	75.0	64.7
PyN-7	54.5	61.1	61.1	75.0	75.0	125.0	133.3	100.0	80.0	68.2	63.6	68.2	57.9
Mean	53.3	60.3	60.9	65.7	84.1***	107.9***	127.4***	102.9***	75.3***	65.8	63.7	78.3***	59.9
SD	3.2	1.7	2.3	5.3	12.4	16.2	11.5	10.4	7.9	2.3	1.7	10.5	2.4

***, $p<0.001$

Table S9: Sequential analysis of MPV value, related to Fig. 4b(vi)

After infection	MPV value (fL)												
	day 0	day 2	day 4	day 7	day 10	day 14	day 17	day 21	day 28	day 35	day 42	day 49	day 56
PyN-1	7.3	6.8	10.4	7.9	9.4	OOR	13.3	OOR	7.8	7.4	8.4	8.2	7.8
PyN-2	7.2	7.4	OOR	8.5	8.9	OOR	10.0	11.7	8.5	8.1	7.7	7.6	7.4
PyN-3	7.2	6.8	8.6	8.9	9.7	11.9	OOR	9.9	7.6	7.3	8.7	7.8	6.9
PyN-4	8.1	7.1	8.1	9.2	OOR	9.9	10.1	11.4	7.3	7.2	7.2	7.1	7.3
PyN-5	7.1	7.4	7.9	8.2	9.1	10.0	12.8	OOR	8.3	8.0	7.0	7.7	7.1
PyN-6	7.2	6.7	7.4	8.7	9.1	9.8	OOR	OOR	8.4	8.5	7.5	7.3	7.0
PyN-7	7.1	7.1	7.3	8.7	8.0	10.4	OOR	OOR	8.6	7.6	7.2	6.8	7.1
Mean	7.3	7.0	-	8.6**	-	-	-	-	8.1	7.7	7.7	7.5	7.2
SD	0.3	0.3	-	0.4	-	-	-	-	0.5	0.4	0.6	0.4	0.3

**, $p<0.01$

OOR, out of range

Table S10: Sequential analysis of parasitemia and the parameters after treatment with artemisinin, related to Fig. 5

		After infection (day)			
		0	2	4	7
Parasitemia (%)	Artemisinin-1	7.0	0.27	0.0050	0.0029
	Artemisinin-2	7.7	0.33	0.026	0.0045
	Artemisinin-3	11.7	0.36	0.033	0.0052
	Solvent-1	6.4	8.2	15.8	37.5
	Solvent-2	6.3	7.2	14.4	43.4
	Solvent-3	7.1	7.4	16.5	44.3
RBC count ($10^6/\mu L$)	Artemisinin-1	8.1	6.1	9.6	10.6
	Artemisinin-2	8.7	7.3	8.4	10.5
	Artemisinin-3	9.7	7.2	8.0	10.0
	Solvent-1	9.0	5.8	5.4	3.7
	Solvent-2	9.1	5.2	5.1	4.3
	Solvent-3	9.3	6.2	4.5	3.4
WBC count ($10^3/\mu L$)	Artemisinin-1	1.8	7.3	6.3	7.7
	Artemisinin-2	2.7	4.7	5.3	6.5
	Artemisinin-3	3.8	10.0	4.5	7.2
	Solvent-1	2.3	13.9	13.8	7.3
	Solvent-2	6.3	20.6	14.0	5.8
	Solvent-3	5.5	11.9	16.3	7.7
PLT count ($10^5/\mu L$)	Artemisinin-1	2.5	6.9	7.5	8.3
	Artemisinin-2	4.1	5.2	6.6	8.8
	Artemisinin-3	3.0	5.8	6.9	13.2
	Solvent-1	3.0	3.5	5.8	2.2
	Solvent-2	2.5	5.4	5.0	1.4
	Solvent-3	2.8	3.1	5.5	2.2
HCT value (%)	Artemisinin-1	45.0	35.0	50.0	55.0
	Artemisinin-2	50.0	40.0	46.7	55.0
	Artemisinin-3	55.0	40.0	45.0	50.0
	Solvent-1	48.3	30.0	33.3	28.3
	Solvent-2	48.3	28.8	30.0	35.0
	Solvent-3	50.0	33.8	26.7	26.7
MCV value (fL)	Artemisinin-1	56.3	58.3	52.6	51.6
	Artemisinin-2	57.7	55.8	54.9	52.4
	Artemisinin-3	56.9	57.1	56.3	50.0
	Solvent-1	53.7	51.1	60.6	77.4
	Solvent-2	53.7	54.8	60.0	81.0
	Solvent-3	53.6	55.1	59.3	76.2
MPV value (fL)	Artemisinin-1	7.5	7.7	7.0	7.2
	Artemisinin-2	7.7	7.6	7.9	7.3
	Artemisinin-3	7.9	7.3	8.0	7.0
	Solvent-1	8.1	8.5	8.3	9.9
	Solvent-2	8.0	8.6	8.6	9.5
	Solvent-3	7.6	8.2	8.6	9.4
